# Characterization of Two New CTX-M-25-Group Extended-Spectrum β-Lactamase Variants Identified in *Escherichia coli* Isolates from Israel

**DOI:** 10.1371/journal.pone.0046329

**Published:** 2012-09-26

**Authors:** Jascha Vervoort, Anna Baraniak, Muriel Gazin, Julia Sabirova, Christine Lammens, Meital Kazma, Anna Grabowska, Radosław Izdebski, Yehuda Carmeli, Samir Kumar-Singh, Marek Gniadkowski, Herman Goossens, Surbhi Malhotra-Kumar

**Affiliations:** 1 Department of Medical Microbiology, Universiteit Antwerpen, Antwerp, Belgium; 2 Vaccine and Infectious Disease Institute, Universiteit Antwerpen, Antwerp, Belgium; 3 Department of Molecular Microbiology, National Medicines Institute, Warsaw, Poland; 4 Division of Epidemiology and Laboratory for Molecular Epidemiology and Antibiotic Research, Tel Aviv Sourasky Medical Center, Sackler Faculty of Medicine, Tel Aviv University, Tel Aviv, Israel; 5 Molecular pathology Group, Cell Biology and Histology, Universiteit Antwerpen, Antwerp, Belgium; University of Massachusetts Medical School, United States of America

## Abstract

**Objectives:**

We characterized two new CTX-M-type extended-spectrum β-lactamase (ESBL) variants in *Escherichia coli* isolates from stool samples of two elderly patients admitted at the Tel Aviv Sourasky Medical Center, Israel. Both patients underwent treatment with cephalosporins prior to isolation of the *E. coli* strains.

**Methods:**

ESBLs were detected by the double-disk synergy test and PCR-sequencing of β-lactamase genes. The *bla*_CTX-M_ genes were cloned into the pCR-BluntII-TOPO vector in *E. coli* TOP10. The role of amino-acid substitutions V77A and D240G was analyzed by site-directed mutagenesis of the *bla*_CTX-M-94_ and *bla*_CTX-M-100_ genes and comparative characterization of the resulting *E. coli* recombinants. MICs of β-lactams were determined by Etest. Plasmid profiling, mating experiments, replicon typing and sequencing of *bla*_CTX-M_ flanking regions were performed to identify the genetic background of the new CTX-M variants.

**Results:**

The novel CTX-M β-lactamases, CTX-M-94 and -100, belonged to the CTX-M-25-group. Both variants differed from CTX-M-25 by the substitution V77A, and from CTX-M-39 by D240G. CTX-M-94 differed from all CTX-M-25-group enzymes by the substitution F119L. Glycine-240 was associated with reduced susceptibility to ceftazidime and leucine-119 with increased resistance to ceftriaxone. *bla*_CTX-M-94_ and *bla*_CTX-M-100_ were located within IS*Ecp1* transposition units inserted into ∼93 kb non-conjugative IncFI and ∼130 kb conjugative IncA/C plasmids, respectively. The plasmids carried also different class 1 integrons.

**Conclusions:**

This is the first report on CTX-M-94 and -100 ESBLs, novel members of the CTX-M-25-group.

## Introduction

CTX-Ms are the most prevalent extended-spectrum β-lactamases (ESBLs) in Enterobacteriaceae causing hospital- and community-acquired infections [1]–[3]. The *bla*_CTX-M_ genes are usually located on plasmids, and are derivatives of chromosomal β-lactamase genes of the *Kluyvera* genus due to multiple mobilization events [1], [4]. Usually, these plasmids easily spread in microbial populations, also carrying other resistance genes such as those coding for aminoglycoside acetyltransferases, dihydropteroate synthases or other β-lactamases [3]. New CTX-M variants arise rapidly and so far 131 different enzymes of this family have been identified (http://www.lahey.org/studies). They can all be assigned to five different subfamilies, based on amino acid identity: the CTX-M-1, -2, -8, -9, and -25 groups [1]. New CTX-M variants within these groups emerge by the gradual accumulation of mutations, some of which affect enzyme activity and resistance phenotype, and are being selected by antibiotic pressure [5], [6]. Unlike the CTX-M-1, -2 and -9 groups, the CTX-M-25-like β-lactamases have been rarely observed worldwide [7]–[9]. However, the situation in Israel is in stark contrast as a remarkable number of CTX-M-25-group enzymes, namely CTX-M-25, -26, 39 and -41, have been previously reported in there [10], [11]. We recently identified two new CTX-M-25-group variants, CTX-M-94 and -100, in *Escherichia coli* isolates from patients admitted at the Tel Aviv Sourasky Medical Center, Israel. The aim of this study was to characterize CTX-M-94 and -100 and to understand the phylogenetic relationships between the novel and the known CTX-M-25-group variants.

## Methods

### Bacterial Strains

Two *E. coli* clinical strains were recovered in 2008–2009 during an epidemiological study from screening stool samples of two elderly patients hospitalized at the Tel Aviv Sourasky Medical Center, Israel. These isolates were identified using mass spectrometry (MALDI-TOF, Bruker Daltonics, Bremen, Germany). *E. coli* TOP10 electrocompetent cells (Invitrogen, Carlsbad, CA, USA) were utilized as hosts for cloning and site-directed mutagenesis experiments, and rifampicin-resistant *E. coli* A15 was used as a recipient in mating tests [12].

### Phenotypic ESBL Detection and Susceptibility Testing

The ESBL phenotype of the two clinical isolates was confirmed by the double-disk synergy test using cefotaxime, ceftazidime, cefepime and amoxicillin/clavulanate disks [13]. Susceptibility to β-lactam antibiotics (amoxicillin, amoxicillin/clavulanate, ampicillin, aztreonam, cefepime, cefotaxime, cefoxitin, ceftazidime, ceftazidime/clavulanate, ceftriaxone, imipenem, piperacillin, and piperacillin/tazobactam) was tested by disk diffusion (disks from Oxoid, Basingstoke, UK) and by Etest (bioMérieux, Marcy l'Etoile, France) according to the CLSI guidelines [14].

### Genotypic ESBL Detection and bla_CTX-M_ Characterization

Total DNA was extracted from the isolates by alkaline lysis as described previously [15]. Plasmid DNA was obtained from the isolates with the PureLink HiPure Plasmid Miniprep Kit according to the manufacturer’s protocol (Invitrogen). β-lactamase genes *bla*_CTX-M_, *bla*_SHV_, and *bla*_TEM_ were detected by PCR using universal primers with modified cycling conditions: 10 min at 95°C, 30 cycles of 30s at 95°C, 30s at 59°C, and 1 min at 72°C, and 10 min at 72°C [16]–[18]. The entire *bla*_CTX-M-94_ and *bla*_CTX-M-100_ genes were amplified using previously described primers CTX-M-25-F (specific for the IS*Ecp1* element) and CTX-M-25-R primers with modified cycling conditions: 10 min at 95°C, 30 cycles of 30 s at 95°C, 30 s at 55°C, and 1.5 min at 72°C, and 10 min at 72°C [10]. The amplicons were sequenced directly on both strands, compared to known *bla*_CTX-M_ sequences (GenBank and Lahey Clinic), and analyzed using BLASTn (NCBI, http://www.ncbi.nlm.nih.gov) and the Lasergene software (DNASTAR, Madison, WI, USA). The extrachromosomal location of *bla*_CTX-M-94_ and *bla*_CTX-M-100_ was confirmed by amplification from plasmid DNA in triplicate, as well as by plasmid electroporation into *E. coli* TOP10.

### AmpC Detection

Production of AmpC-like β-lactamases was assessed by the combination disk test with cefotaxime and ceftazidime disks, with and without phenylboronic acid (20 µl of 20 mg/mL solution per disk). A≥5 mm increase in zone diameter after addition of boronate indicated the higher-level expression of AmpC. Genes coding for acquired AmpC types were identified by PCR-sequencing analysis on plasmid DNA with target-specific primer pairs [19].

### Strain Typing and Phylogrouping

Sequence types (STs) of the *E. coli* strains were determined by multilocus sequence typing (MLST) [20], using the MLST database at the ERI, University College Cork (http://mlst.ucc.ie/mlst/dbs/Ecoli). Phylogenetic grouping was performed by PCR [21].

### Detection of Enzymatic Activity and Isoelectric Focusing of β-lactamases

The presence of functional β-lactamases was evidenced by a qualitative method utilizing the chromogenic cephalosporin nitrocefin (Calbiochem, Merck Chemicals, Nottingham, UK). Hydrolysis of nitrocefin by β-lactamases results in a distinct colour shift from yellow (λ_max_ = 390 nm at pH 7.0) to red (λ_max_ = 486 nm at pH 7.0). A nitrocefin solution (500 mg/L) was added to crude bacterial sonicates to show presence of functional β-lactamases. Sonicates positive for β-lactamase activity were subjected to isoelectric focusing (IEF) as described previously [12], in a Model 111 Mini IEF Cell (Bio-Rad, Hercules, CA, USA) The IEF gel was homogeneously covered with the nitrocefin solution to mark all bands corresponding to the different β-lactamases present in the bacterial cell extracts.

### Cloning of bla_CTX-M_ genes

Blunt-ended amplicons consisting of *bla*_CTX-M_ genes and ∼30 bp upstream regions were produced using Platinum Pfx DNA Polymerase (Invitrogen) and the primer pair CTX-M-25-F/R with modified cycling conditions: 10 min at 94°C, 30 cycles of 15 s at 94°C, 30 s at 55°C, and 1.5 min at 68°C, and 10 min at 68°C. PCR products were cloned into the pCR-BluntII-TOPO vector (Invitrogen), electrotransformed into *E. coli* TOP10, and selected on LB agar supplemented with kanamycin (50 mg/L) and cefotaxime (2 mg/L). Transformants (pCR-CTX-M-94 and -100) were screened for presence of the *bla*_CTX-M_ genes by PCR and sequencing using M13 primers according to the manufacturer’s protocol.

### Site-directed Mutagenesis

Single point mutations were introduced into pCR-CTX-M-94 and -100 with the QuikChange II Site-Directed Mutagenesis Kit (Stratagene, La Jolla, CA, USA) according to the manufacturer’s protocol. Mutagenic primer pairs (available on request) were designed to replace alanine at position 77 with valine (A77V) and glycine at position 240 with aspartic acid (G240D). The mutagenized pCR-CTX-M-94 and -100 plasmids were electrotransformed into *E. coli* TOP10 and their susceptibility profiles to β-lactams were screened by Etest.

### Plasmid Typing

Total plasmid DNA obtained from the clinical isolates was electroporated into *E. coli* TOP10 to separate coexisting multiple plasmids. Unique plasmids were identified by screening the transformants for presence of *bla* genes, including *bla*_CTX-M_, *bla*_TEM_ and *bla*_CMY-2_, by PCR. Plasmid profiles of the transformants carrying unique plasmids were determined by restriction digestion of their plasmid DNA with *HpaI* (New England Biolabs, Ipswich, MA, USA). Restriction products were separated along with a 1 Kb DNA Extension Ladder (Invitrogen) on a 0.7% agarose gel for 10 h at 2.5 V/cm with 0.5x Tris-borate-EDTA buffer. Plasmid sizes were estimated by relative mobility calculations. Presence of *bla* genes was confirmed by hybridization with *bla*_CTX-M-25-_group-_,_ *bla*_TEM_- and *bla*_CMY-2_-specific DIG-labeled probes and a DIG Luminescent Detection Kit (Roche Applied Science, Indianapolis, IN, USA) according to the manufacturer’s protocols. Replicon types of all unique plasmids were identified by PCR-based replicon typing as described previously [22]. Conjugation was carried out as described previously [12], utilizing the clinical strains producing CTX-M-94 and -100 as donors and rifampicin-resistant *E. coli* A15 strain as a recipient. Transconjugants were selected on LB agar with cefotaxime (2 mg/L) and rifampicin (128 mg/L), and confirmed by the phenotypic disk tests, PCR of *bla* genes, and MLST against the donors and recipient. Transfer efficiencies were calculated based on the ratio of CFUs/mL of transconjugants per donor cell.

### Nucleotide Sequence Accession Numbers

*bla*_CTX-M-94_ and *bla*_CTX-M-100_ nucleotide sequences were assigned GenBank accession numbers HM167760 and FR682582, respectively.

## Results and Discussion

### E. coli Isolates Harbouring Novel CTX-M Variants

We recovered the CTX-M-94 and -100-producing *E. coli* strains from screening stool samples of two elderly hospitalized patients. The patients received various antibiotics, including cephalosporins (ceftriaxone), β-lactam/inhibitor combinations (piperacillin/tazobactam), cephamycins and carbapenems (imipenem). The CTX-M-94-producing *E. coli* belonged to ST131 and phylogroup B2. ST131 is a uropathogenic clone that has spread worldwide, usually associated with ESBLs, in particular with CTX-M-15 of the CTX-M-1 group [3], [23], [24]. Along with CTX-M-94, the *E. coli* isolate also harboured two other acquired β-lactamases, TEM-1 and the AmpC-like cephalosporinase CMY-2. The CTX-M-100-producing *E. coli* belonged to ST88 (clonal complex ST23) and phylogroup A. Strains belonging to phylogroup A are generally more common in the intestinal flora, and both the complex ST23 and the clone ST88 belong to the globally spread members of the phylogroup [25]. The CTX-M-100-harbouring isolate did not express any other acquired β-lactamase.

MICs of β-lactams for the CTX-M-94- and -100-producing isolates are shown in Table 1. The CTX-M-94 harbouring isolate exhibited four- to approximately 200-fold higher MICs of amoxicillin/clavulanate, cefoxitin, cefotaxime, ceftriaxone, ceftazidime (alone and with clavulanate), cefepime and aztreonam, when compared to the CTX-M-100 producer. According to the current CLSI guidelines [14], both isolates were highly resistant to amoxicillin, ampicillin, piperacillin, cefotaxime and ceftriaxone, and susceptible to piperacillin/tazobactam, cefepime and imipenem. The resistance pattern of the CTX-M-100-producing isolate was similar to that of the previously described CTX-M-39-producing *E. coli* from the same hospital [11], and matched well the typical CTX-M-associated phenotype, with asymmetry in MICs of cefotaxime and ceftazidime and good activity of inhibitor combinations [1], [5], [26]. In contrast, resistance of the CTX-M-94 producer was largely influenced by CMY-2, both in level and type. AmpC β-lactamases like CMY-2 are known to hydrolyze cephamycins and are much less inhibited by class A enzyme inhibitors compared to ESBLs [27], as exemplified by high MICs of cefoxitin and clavulanate combinations, respectively (Table 1). Furthermore, AmpC β-lactamases are also capable of hydrolyzing oxyiminocephalosporins and monobactams, as exemplified by elevated MICs of cefotaxime, ceftazidime, ceftriaxone and aztreonam (Table 1). Finally, the influence of CMY-2 on the phenotype exhibited by the wild-type CTX-M-94-harbouring isolate was confirmed by the decreased resistance pattern of pCR-CTX-M-94, a recombinant *bla*_CTX-M-94_-harbouring strain that lacked CMY-2. pCR-CTX-M-94 showed a major decrease in the MICs of cefoxitin (>256 vs. 2 mg/L), ceftazidime/clavulanate (>4 vs. 0.25 mg/L) and cefotaxime (>256 mg/L vs. 16 mg/L), and a more moderate decrease in the MICs of ceftriaxone (>256 vs. 48 mg/L), ceftazidime (12–16 vs. 4 mg/L) and amoxicillin/clavulanate (8 vs. 3 mg/L).

**Table 1:** MICs of β-lactams of the CTX-M-94- and -100-producing *E. coli*, *E. coli* TOP10 transformed with wild-type pCR-CTX-M-94 and -100 and mutagenized pCR-CTX-M-94 and -100.

Antibiotics	Minimum inhibitory concentrations (mg/L)							
	*E. coli*	*E. coli*	pCR-CTX-M-94	pCR-CTX-M-94	pCR-CTX-M-94	pCR-CTX-M-100	pCR-CTX-M-100	pCR-CTX-M-100
	(clinical isolate)	(clinical isolate)	(wild type)	(mutagenized)	(mutagenized)	(wild type)	(mutagenized)	(mutagenized)
	(CTX-M-94, TEM-1, CMY-2)	(CTX-M-100)	(A77, L119, G240)	(V77, L119, G240)	(A77, L119, D240)	(A77, F119, G240)	(V77, F119, G240)	(A77, F119, D240)
							( = CTX-M-25)	( = CTX-M-39)
Amoxicillin	>256*	>256*	>256*	>256*	>256*	>256*	>256*	>256*
Amoxicillin/clavulanate	8**	2	3	3	3	3	3	3
Ampicillin	>256*	>256*	>256*	>256*	>256*	>256*	>256*	>256*
Aztreonam	6**	2	4	4	1.50	4	4	1.50
Cefepime	8	1.50	2	2	1.50	2	2	1
Cefotaxime	>256*	12*	16*	16*	4*	16*	24*	1.50**
Cefoxitin	>256*	1.50	2	4	1	2	4	1.50
Ceftazidime	12–16**	1.50	4	4	0.50	3	3	0.38
Ceftazidime/clavulanate	>4	0.06	0.25	0.25	0.19	0.25	0.25	0.19
Ceftriaxone	>256*	8*	48*	24*	12*	12*	32*	4*
Imipenem	0.75–1.50	1.50	0.38	0.38	0.38	0.25	0.25	0.38
Piperacillin	>256*	>256*	>256*	>256*	>256*	>256*	>256*	64**
Piperacillin/tazobactam	0.75	0.50	1.50	1	1	1.50	2	1.50

* Indicates high-level resistance.

** Indicates intermediate resistance.

Other differences in resistance could be attributed to molecular differences between CTX-M-94 and -100 enzymes, which are discussed in the following sections.

### Molecular Characterization of Novel CTX-M Variants

**Figure 1:**
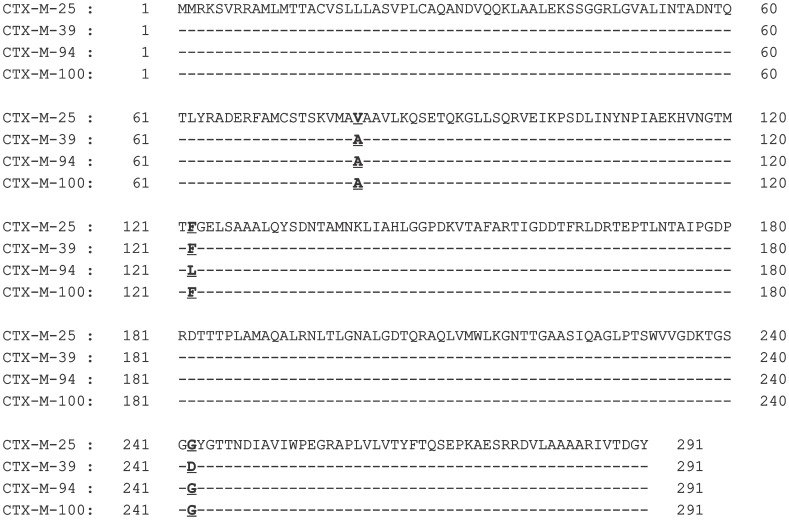
Comparison of the amino acid sequences of the novel CTX-M-25-group variants (CTX-M-94 and -100), with the known CTX-M-25-group enzymes (CTX-M-25 and -39). Differences are highlighted in bold.

**Figure 2:**
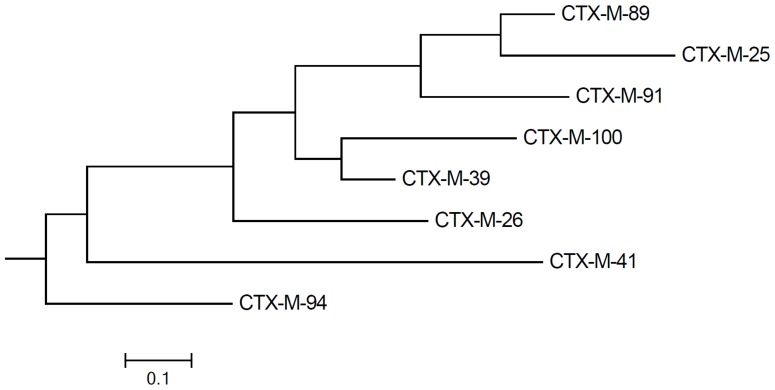
Phylogenetic tree of CTX-M-25-group subtypes. Scale: amino acid substitutions per 100 residues.

CTX-M-94 and -100 belonged to the CTX-M-25-group, sharing >99% homology with CTX-M-25 and -39 (Figure 1). Both variants differed from CTX-M-25 by the substitution V77A, and from CTX-M-39 by the substitution D240G. Additionally, CTX-M-94 was remarkable in that it had the substitution F119L (the only difference between CTX-M-94 and -100). This is the first report of a CTX-M-25-group member harbouring leucine-119. Leucine is usually present at position 119 in enzymes belonging to CTX-M-1, -2, -8, and -9 groups, with the exception of CTX-M-11 and -85 that have proline here. Its role in resistance towards β-lactams had not been studied before and was therefore investigated in this study by cloning experiments (see next section). Furthermore, silent nucleotide substitutions were absent in either *bla*_CTX-M-94_ or *bla*_CTX-M-100_ when compared with *bla*_CTX-M-25_ and *bla*_CTX-M-39_. Genetic relatedness of all known CTX-M-25-group enzymes, calculated using MEGA 5 (Figure 2) [28], suggested earlier branching of the CTX-M-94 branch, possibly due to the F119L substitution. It also indicated that CTX-M-100 is most closely related to CTX-M-39. IEF showed double bands, with pI values of 7.3 and 7.5, for both CTX-M-94 and -100 enzymes. A similar pattern has been shown previously for CTX-M-39, with pIs of 6.8 and 7.0, while CTX-M-25 has a pI of 7.5 [8], [10].

### Functional Characterization of Wild-type and Mutagenized CTX-Ms

To determine the resistance patterns conferred by the new CTX-M variants and the specific role of the F119L substitution, *bla*_CTX-M-94_ and *bla*_CTX-M-100_ with upstream regions were cloned in pCR-BluntII-TOPO and expressed in the isogenic background of *E. coli* TOP10 (Table 1). The analysis revealed high-level resistance towards amoxicillin, ampicillin, piperacillin, cefotaxime and ceftriaxone for both CTX-M-94 and -100 producers without significant differences in MIC values, except for a four-fold lower ceftriaxone MIC in the CTX-M-100 producer (12 mg/L) when compared to that with CTX-M-94 (48 mg/L).

To investigate the role of the V77A and D240G substitutions in the sequence background of CTX-M-25-group β-lactamases, reverse mutations were introduced separately into the *bla*_CTX-M-94_ and *bla*_CTX-M-100_ genes. In CTX-M-100, the replacement of alanine-77 with valine and glycine-240 with aspartic acid resulted in a reproduction of the protein sequences of CTX-M-25 and -39, respectively. When compared with CTX-M-94 and -100 wild-type strains, the G240D revertants conferred, as expected, a remarkable decrease in the MICs of ceftazidime (4–3 vs. 0.50–0.38 mg/L), and a more moderate decrease in the MICs of aztreonam (4 vs. 1.50 mg/L), cefotaxime (16 vs. 4–1.50 mg/L) and ceftriaxone (48–12 vs. 12–4 mg/L). In addition, the G240D substitution caused a remarkable decrease in the MICs of piperacillin (>256 vs. 64 mg/L) in CTX-M-100, while having no effect in CTX-M-94. These data fit well with previous crystallographic studies showing that glycine-240, located in the B3 β-strand which lines the active site, is a major contributor to ceftazidime resistance. When aspartic acid at position 240 is replaced by glycine, the mobility of the B3 β-strand is increased, resulting in enhanced protein flexibility and facilitation of hydrolysis of β-lactams with bulkier side chains. However, this increased mobility is not only associated with increased activity, but also with decreased stability of the CTX-M enzyme [5], [29]. A recent *in vitro*-evolution study demonstrated that cefotaxime and ceftazidime probably acted as diversifying agents in the evolution of CTX-M-1 group enzymes [6]. One of the three distinct trajectories analyzed was the D240G route wherein CTX-Ms carrying glycine-240 exhibited moderate increases in ceftazidime MICs, while maintaining high cefotaxime MICs. It was proposed that D240G allows for better adaptation of CTX-M producers to the concurrent exposure to both drugs. Most probably, CTX-M-94 and -100 illustrate this phenomenon in the evolution of the CTX-M-25-group enzymes.

The A77V substitution caused a two-fold increase in cefoxitin MICs (2 vs. 4 mg/L) in CTX-M-94 and-100, and only a minor increase in cefotaxime MICs (16 vs. 24 mg/L) in CTX-M-100. It had no effect on the MICs of ceftazidime. In contrast to our results, Novais *et al.* observed a minor decrease in cefoxitin MICs and a two-fold increase in MICs of cefotaxime and ceftazidime after introducing A77V into CTX-M-15 [6]. These conflicting outcomes might be explained by differences between CTX-M-1- and -25- group enzymes in amino acid residues that line the highly conserved active site. CTX-M-15, a prototypal CTX-M-1 group enzyme, differs from CTX-M-94 and -100 at positions 103 (valine and isoleucine, respectively) and 133 (valine and threonine, respectively) that lie in the α structural domain of the CTX-M enzyme and in close proximity to the Ω loop (based on the active site structure described in Delmas *et al.* [30]. Finally, the A77V substitution caused a two-fold decrease (MICs, 48 vs. 12 mg/L) and a three-fold increase (MICs, 12 vs. 32 mg/L) in ceftriaxone MICs in CTX-M-94 and -100, respectively.

### Plasmid Typing

**Figure 3:**
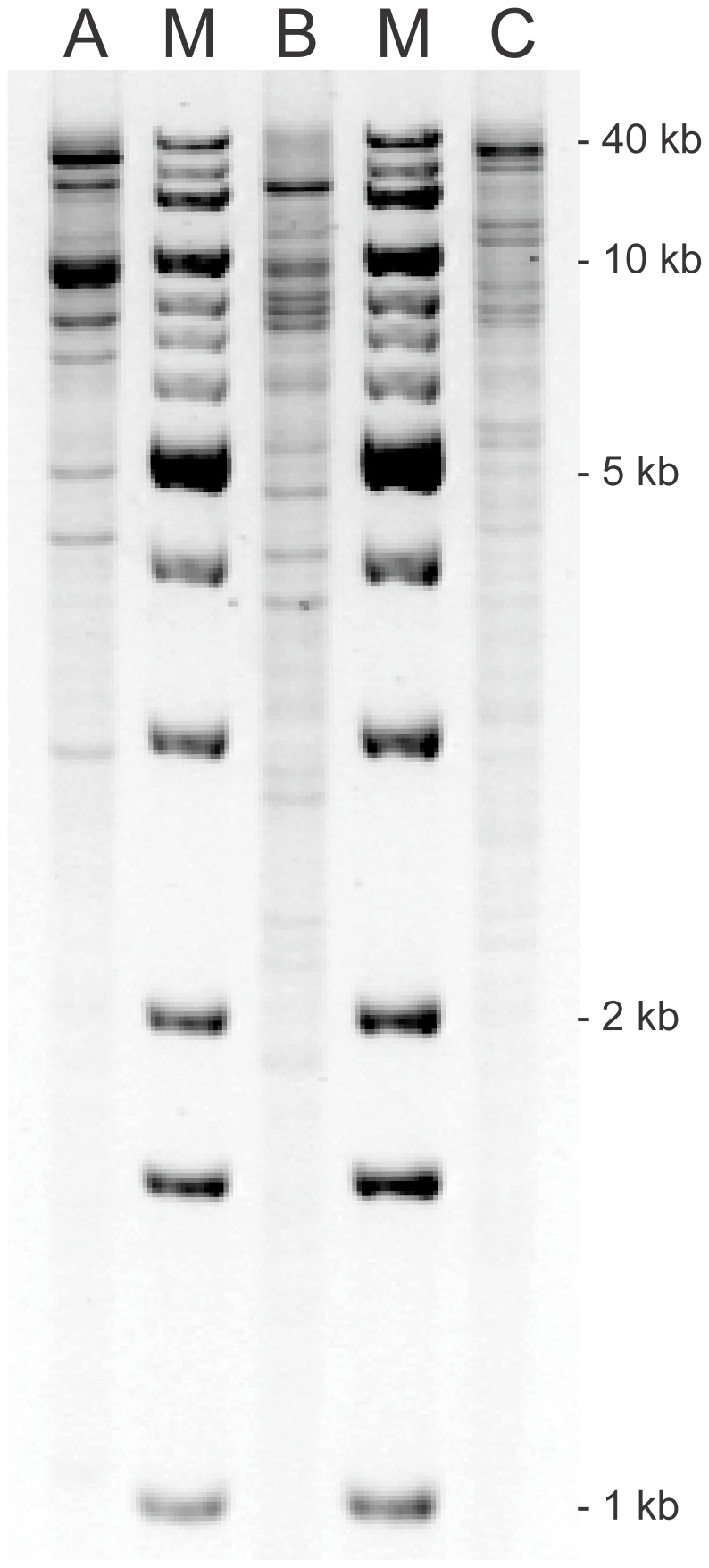
*HpaI* plasmid profiles of *bla*_CTX-M-94_-, *bla*_CMY-2_- and *bla*_CTX-M-100_-harbouring plasmids. A 0.7% agarose gel was loaded with *HpaI* restricted plasmid DNA purified from three recombinant *E. coli* TOP10 strains containing the (A) *bla*_CTX-M-94_-, (B) *bla*_CMY-2_- and (C) *bla*_CTX-M-100_-harbouring plasmids, respectively, and stained with GelRed. Relative mobility calculations, based on known band sizes of the 1 Kb DNA Extension Ladder (M), estimated total plasmid sizes to be (A) 93 kb, (B) 98 kb and (C) 130 kb.

Replicon typing and *HpaI* plasmid profiling (Figure 3) revealed that *bla*_CTX-M-94_ was located on a ∼93 kb IncFI plasmid and *bla*_CTX-M-100_ on a ∼130 kb IncA/C plasmid. The *bla*_CMY-2_ gene, which was also present in the CTX-M-94-producing isolate, was located on a second ∼98 kb IncI1 plasmid. The *bla*_CTX-M-100_-harbouring plasmid was transferred to recipient cells with a high efficiency (5.4×10^−2^ per donor cell). On the other hand, the *bla*_CTX-M-94_-carrying plasmid did not transfer. Instead, the *bla*_CMY-2_-harbouring plasmid was transferred with an efficiency of 3.8×10^−2^ per donor cell.

### Genetic Environment of bla_CTX-M_ Genes

Both *bla*_CTX-M-94_ and *bla*_CTX-M-100_ were located 36 bp downstream of IS*Ecp1*, the most probable factor of mobilization of these genes from the *K. georgiana* chromosome [31], and sequences separating the genes from IS*Ecp1* were identical to each other. Similar distances were previously reported for *bla*_CTX-M-25_ and *bla*_CTX-M-26_ identified in Canada and the UK, respectively [8]. Interestingly, other *bla*_CTX-M-25_-like genes identified in Israel were found to be located 126–128 bp downstream of IS*Ecp1* [11]. These findings suggest that CTX-M-94 and -100 might not have emerged from other CTX-M-25-group members identified in Israel so far, but from an independent IS*Ecp1*-mediated mobilization of a precursor *K. georgiana* gene.

This assumption seems to be further supported by the lack of the direct association of *bla*_CTX-M-94_ and *bla*_CTX-M-100_ with a class 1 integron, demonstrated by a PCR-sequencing based class 1 integron analysis as described previously [11]. Unlike the above mentioned *bla*_CTX-M-25_-like genes identified in Israel, which were inserted into a class 1 integron [11], the IS*Ecp1*-*bla*_CTX-M-94_ and IS*Ecp1*-*bla*_CTX-M-100_ transposition modules were not present in the variable regions of the integrons identified in the *bla*_CTX-M-94_- and *bla*_CTX-M-100_-containing plasmids. These integrons significantly differed from each other. The one present on the plasmid with *bla*_CTX-M-94_ harboured *aacC1* (aminoglycoside acetyltransferase; resistance to gentamicin), *orfX* (hypothetical protein), *orfQ* (hypothetical protein) and *aadA1* (aminoglycoside adenylyltransferase; resistance to spectinomycin and streptomycin) gene cassettes, while the one on the plasmid with *bla*_CTX-M-100_ harboured *aadB* (aminoglycoside adenylyltransferase; resistance to gentamicin/kanamycin/tobramycin), *ereA* (erythromycin esterase type 1; resistance to erythromycin) and *aadA1*. For now, it is unclear whether CTX-M-94 and -100 have emerged from each other or from other CTX-M-25-group members by independent mutational events. Because of limited publicly available sequence data, it is not possible to make more global assumptions on the evolutionary paths of the CTX-M-25-group enzymes.

### Conclusions

This is the first report describing two novel ESBL variants of the CTX-M-25-group, CTX-M-94 and -100, identified in two *E. coli* isolates belonging to different clones, ST131 and ST88, respectively. Both *bla*_CTX-M-94_ and *bla*_CTX-M-100_ were found within IS*Ecp1* transposition units, located on ∼93 kb non-conjugative IncFI and ∼130 kb conjugative IncA/C plasmids, respectively. CTX-M-94 is the first CTX-M-25-group member carrying leucine-119, which was shown here to be associated with increased resistance to ceftriaxone. We have also confirmed the role of the D240G substitution in the increase of enzyme activity against ceftazidime in the context of the CTX-M-25-type enzymes.
